# Brain‐derived neurotrophic factor attenuates doxorubicin‐induced cardiac dysfunction through activating Akt signalling in rats

**DOI:** 10.1111/jcmm.13012

**Published:** 2016-11-07

**Authors:** Pengzhou Hang, Jing Zhao, Li Sun, Minghui Li, Yu Han, Zhimin Du, Yue Li

**Affiliations:** ^1^ Institute of Clinical PharmacologyThe Second Affiliated Hospital (Key Laboratory of Drug Research, Heilongjiang Higher Education Institutions)Harbin Medical UniversityHarbinChina; ^2^ Heilongjiang Academy of Medical ScienceHarbinChina; ^3^ Department of CardiologyThe First Affiliated Hospital (Key Laboratory of Cardiac Diseases and Heart Failure)Harbin Medical UniversityHarbinChina

**Keywords:** BDNF, doxorubicin, apoptosis, Akt, mTOR, Bad

## Abstract

The clinical application of doxorubicin (Dox) is limited by its adverse effect of cardiotoxicity. Previous studies have suggested the cardioprotective effect of brain‐derived neurotrophic factor (BDNF). We hypothesize that BDNF could protect against Dox‐induced cardiotoxicity. Sprague Dawley rats were injected with Dox (2.5 mg/kg, 3 times/week, i.p.), in the presence or absence of recombinant BDNF (0.4 μg/kg, i.v.) for 2 weeks. H9c2 cells were treated with Dox (1 μM) and/or BDNF (400 ng/ml) for 24 hrs. Functional roles of BDNF against Dox‐induced cardiac injury were examined both *in vivo* and *in vitro*. Protein level of BDNF was reduced in Dox‐treated rat ventricles, whereas BDNF and its receptor tropomyosin‐related kinase B (TrkB) were markedly up‐regulated after BDNF administration. Brain‐derived neurotrophic factor significantly inhibited Dox‐induced cardiomyocyte apoptosis, oxidative stress and cardiac dysfunction in rats. Meanwhile, BDNF increased cell viability, inhibited apoptosis and DNA damage of Dox‐treated H9c2 cells. Investigations of the underlying mechanisms revealed that BDNF activated Akt and preserved phosphorylation of mammalian target of rapamycin and Bad without affecting p38 mitogen‐activated protein kinase and extracellular regulated protein kinase pathways. Furthermore, the beneficial effect of BDNF was abolished by BDNF scavenger TrkB‐Fc or Akt inhibitor. In conclusion, our findings reveal a potent protective role of BDNF against Dox‐induced cardiotoxicity by activating Akt signalling, which may facilitate the safe use of Dox in cancer treatment.

## Introduction

Doxorubicin (Dox) is a widely used chemotherapeutic drug in clinical setting 1. Despite successful treatment of various malignancies, Dox‐induced dose‐dependent progressive myocardial damage seriously limits its clinical usage 2. Doxorubicin‐induced cardiotoxicity is characterized by oxidative stress, DNA damage, cardiomyocyte apoptosis and interstitial fibrosis *et al*. 3, 4, 5. During the past years, many studies were performed to explore the methods for attenuating Dox‐induced cardiac dysfunction 6. Several agents have been uncovered to protect against the cardiotoxicity of Dox such as angiotensin converting enzyme inhibitors (enalapril) 7, β‐blockers (metoprolol, carvediol) 8, 9 and resveratrol 10, 11. Recently, other new measures were developed to mitigate Dox‐induced cardiomyopathy 12, 13. For example, mesenchymal stem cells produced promising cardioprotective effects against Dox‐induced damage by paracrine of potent cytokines such as migration inhibitory factor and growth differentiation factor‐15 12. However, the direct effects of those cytokines/growth factor on Dox‐induced cardiomyopathy remain largely unknown.

Brain‐derived neurotrophic factor (BDNF) is one of the most abundant neurotrophins in the mammalian central nervous system that promotes neuronal survival and differentiation 14. Growing studies have documented that BDNF plays crucial roles in the cardiovascular system 15, 16, 17, 18. Among them, two recent studies proved that BDNF modulated the contraction of normal heart 16, 17. Studies by other laboratories and ours demonstrated BDNF could repair injured myocardium by ameliorating endothelial function and inhibiting cardiomyocyte apoptosis 18, 19. Previous studies also found that BDNF repressed neuroblastoma cells from chemotherapy‐induced apoptosis by activating its receptor tropomyosin receptor kinase B (TrkB) 20, 21. However, whether BDNF could attenuate Dox‐induced cardiotoxicity remains unclear.

PI3K/Akt and mitogen‐activated protein kinase (MAPK) are two key intracellular signalling transduction pathways, which participate in various important biological activities such as apoptosis and autophagy 22, 23. Previous studies have illustrated that activation of Akt and downstream signals, for example, mammalian target of rapamycin (mTOR) and Bad and/or inhibition of p38 MAPK are capable of inhibiting Dox‐induced cardiac injury 24, 25, 26. Therefore, this study aimed to investigate determine the therapeutic role of BDNF against Dox‐induced cardiotoxicity by regulating Akt or MAPK signals to facilitate their safe use in cancer treatment.

## Materials and methods

### Animals

All animal experiments were approved by the Experimental Animal Ethic Committee of Harbin Medical University and conformed to the Guide for the Care and Use of Laboratory Animals published by the US National Institutes of Health (Publication, 8th Edition, 2011). Healthy male Sprague Dawley (SD) rats (weight 220 ± 20 g) were provided by Animal Center of the Second Affiliated Hospital of Harbin Medical University, Harbin, China. The rats have free access to standard rat chow and water and 15 rats were randomly divided into three groups: sham, Dox and BDNF treatment group (5 rats in each group). In Dox group, rats were injected with Dox (2.5 mg/kg, 3 times/week, intraperitoneally) for 2 weeks. In BDNF group, rats were injected with Dox (2.5 mg/kg, 3 times/week, 2 weeks, intraperitoneally) and recombinant human BDNF (0.4 μg/kg/day) every day for 2 weeks *via* tail vein. The dose of BDNF referred to previous study 27. For sham rats, equivalent volume of saline was given. All rats were killed at the end of the 2‐week treatments after detecting cardiac function, and the hearts were quickly excised in cold buffer. Both left and right ventricles were collected and stored at −80°C for subsequent histological, biochemical and Western blot analysis.

### Transmission electron microscopy

Left ventricles were fixed in 2.5% glutaraldehyde and then rinsed in buffer, post‐fixed in PBS 1% OsO4 for 2 hrs. Then, they were stained by 1% uranyl acetate, dehydrated in graded ethanol and embedded in epoxy resin. The sections were electron‐stained and observed by electron microscope (JEM‐1200; JEOL Ltd., Tokyo, Japan).

### Haematoxylin and eosin and Masson trichrome staining

Both left and right ventricular samples were embedded in paraffin and cut into 5 μm thick sections. Then, they were stained with haematoxylin and eosin or Masson trichrome for histological and collagen analysis. The interstitial fibrotic areas were calculated by image analysis software (Image‐pro plus 6.0; Meida Cybernetics LP, Washington, USA). Collagen volume fraction (CVF) was calculated as the ratio of collagen area to total area.

### Echocardiography

Cardiac function was measured by Acuson Sequoia 512 Ultrasound System (Siemens Medical Solutions USA, Inc., Mountain View, CA, USA). Left ventricular end systolic volume (LVESV) and left ventricular end diastolic volume (LVEDV) were measured, and left ventricular ejection fraction (LVEF) and fractional shortening (FS) were calculated.

### Measurement of superoxide dismutase, malondialdehyde and glutathione levels

The concentrations of three enzymes including superoxide dismutase (SOD), malondialdehyde (MDA) and reduced glutathione (GSH) in both left and right ventricular homogenate were determined using commercial kits (Nanjing Jiancheng Bioengineering Institute, Nanjing, China).

### Cell culture

H9c2 cells were cultured in DMEM (high glucose; Corning, Manassas, VA, USA) supplemented with 10% foetal bovine serum and 1% penicillin and streptomycin. The cells were maintained under a water‐saturated atmosphere of 95% air and 5% CO_2_ at 37°C. The cells were exposed to Dox at different concentrations range from 0.3, 1 to 3 μM for different time‐points (6, 24 or 48 hrs), respectively. Recombinant human BDNF (200 and 400 ng/ml) and/or BDNF scavenger TrkB‐Fc (4 μg/ml) were pre‐treated 30 min. before Dox treatment in the presence or absence of Akti (0.5 μM). The effects of Akti and TrkB‐Fc in this concentration have been validated in previous work 19, 28.

### Cell counting kit‐8 assay

Cell viability was determined by cell counting kit‐8 (CCK‐8) kit as described in the manufacturer's protocol (Dojindo, Kumamoto, Japan). After stimulation as designated, the supernatant was removed and 100 μl of DMEM medium containing 10 μl CCK‐8 was added. Then, the plate was incubated for 2 hrs in the incubator. The optical density values were read at 450 nm using an Infinite M200 microplate reader (Tecan, Salzburg, Austria). For each sample, five parallel experimental wells were used to evaluate the cell viability.

### TUNEL assay

*In situ* Cell Death Detection Kit (TUNEL fluorescence FITC kit; Roche, Indianapolis, USA) was used to examine the apoptosis in rat ventricles and H9c2 cells. After washing with PBS for three times, treated samples were fixed by 4% paraformaldehyde for 1 hr, permeabilized in 0.1% Triton x‐100 sodium citrate buffer for 2 min. The nuclei were stained with DAPI (1:30; Beyotime, Haimen, China). Fluorescence staining of ventricles was viewed by Olympus BX‐60 microscope (Tokyo, Japan) while fluorescence staining of cells was scanned by a laser scanning confocal microscope (FV1000; Olympus, Tokyo, Japan). Five high power fields were randomly selected and analysed. The ratio of TUNEL‐positive cells per field was calculated by Olympus Fluoview version 2.0a Viewer software.

### Assessment of DNA damage

DNA damage was evaluated according to previous study 29. After exposure to Dox in the presence or absence of BDNF, H9c2 cells were fixed in 4% paraformaldehyde solution and probed with anti‐γH2AX antibody (Ser139, 20E3; Cell Signaling Technology, Danvers, MA, USA) overnight at 4°C followed by fluorescence‐labelled secondary antibody. Immunofluorescence images were captured by a fluorescence microscope (Zeiss, Oberkochen, Germany).

### Flow cytometry

Annexin‐V‐FLUOS kit (Roche) was used with procedures described in previous studies 30. Briefly, H9c2 cells (1 × 10^6^ cells/ml) were washed twice with cold PBS and resuspended in 500 μl of binding buffer (10 mM HEPES/NaOH [pH 7.4], 140 mM NaCl, 2.5 mM CaCl_2_). A total of 5 μl of AnnexinV‐FITC and 10 μl of 20 μg/ml PI were then added to the cells, which were analysed with a flow cytometer (BD FACSCanto II, BD Biosciences, San Jose, CA, USA). Viable cells were negative for both PI and Annexin V, while apoptotic cells were positive for Annexin V and negative for PI. Late apoptotic dead cells showed both Annexin V and PI positivity. The apoptotic rate was calculated by BD FACSDiva software.

### Western blot

Total protein was extracted and separated from both ventricles and H9c2 cells for immunoblotting analysis with the procedures described in our previous work 19. Membranes were incubated with primary antibodies against BDNF (1:500), TrkB (1:500), p‐Akt (1:1000), t‐Akt (1:1000), p‐mTOR (1:1000), mTOR (1:1000), p‐Bad (1:500), Bad (1:1000), p‐ERK (1:2000), extracellular regulated protein kinase (ERK; 1:1000), p‐p38 (1:1000), p38 (1:1000) or GAPDH (1:1000) overnight at 4°C, followed by incubation with a fluorescence‐labelled secondary antibody (LI‐COR Biosciences, Lincoln, NE, USA). The images were scanned on the Odyssey CLx Infrared Imaging System (LI‐COR Biosciences). Western blot bands were quantified using Odyssey CLx v2.1 software and normalized to GAPDH as an internal control.

### Reagents

Anti‐BDNF and anti‐TrkB antibodies were bought from Abclonal (Wuhan, China). Anti‐p‐Akt (Ser473), anti‐t‐Akt, anti‐p‐mTOR, anti‐mTOR, anti‐p‐Bad (Ser136), anti‐Bad, anti‐p‐ERK (Thr202/Tyr204), anti‐ERK, anti‐p‐p38 (Thr180/Tyr182), anti‐p38 and anti‐γH2AX (Ser139, 20E3) antibodies were provided by Cell Signaling Technology. Anti‐GAPDH antibody was obtained from Kangchen Biotech Co Ltd (Shanghai, China). Doxorubicin was provided by Aladdin Industrial Company (Shanghai, China, purity 98%). Recombinant human BDNF and TrkB‐Fc were purchased from R&D Systems (Minneapolis, MN, USA). Akt inhibitor (Akti) was provided by Sigma‐Aldrich (St. Louis, MO, USA).

### Statistical analysis

All value were presented as mean ± S.E.M. and analysed by SPSS17.0 software. Student's *t*‐test was performed for two‐group comparisons. One‐way anova followed by Dunnet's *t*‐test was used for multiple‐group comparisons. *P* < 0.05 was considered significant.

## Results

### Protein expression of BDNF/TrkB axis in Dox and BDNF‐treated rat ventricles

**Figure 1:**
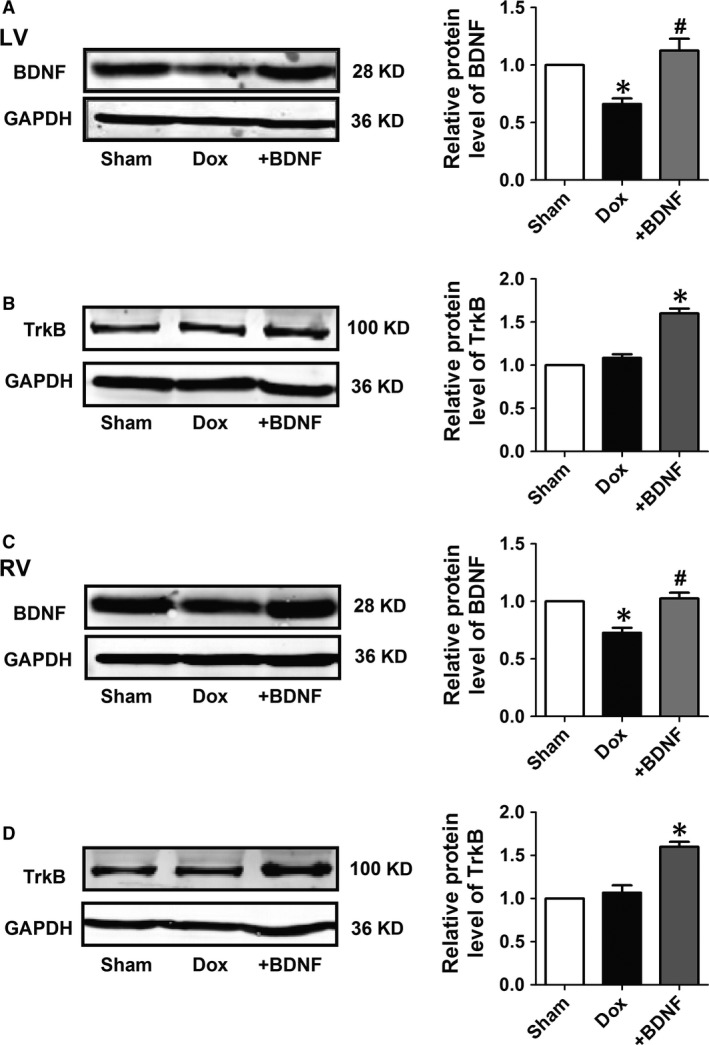
Protein expression of brain‐derived neurotrophic factor (BDNF) and tropomyosin‐related kinase B (TrkB) in Doxorubicin (Dox) and BDNF‐treated rat ventricles. (**A** and **C**) Representative western blot bands and statistical results of BDNF protein expression normalized by GAPDH in left and right ventricles. (**B** and **D**) Representative western blot bands and statistical results of TrkB protein expression normalized by GAPDH in left and right ventricles. *P < 0.05 *versus* sham, #*P* < 0.05 *versus* Dox, *n* = 5 each group.

Protein expression of BDNF and its receptor TrkB were detected in both left and right ventricles to observe the change in cardiac BDNF/TrkB axis in Dox‐treated myocardium and validate the efficiency of BDNF supplement. Figure 1A indicated that protein expression of BDNF was decreased in left ventricles of Dox‐treated rats, which was significantly restored after BDNF administration. Meanwhile, protein expression of TrkB was markedly increased by BDNF, although no significance was found between sham and Dox‐treated rats (Fig. 1B). Similar results of BDNF and TrkB expression were obtained in right ventricles (Fig. 1C and D). Thus, cardiac BDNF was down‐regulated by Dox and was successfully reversed after BDNF injection. Brain‐derived neurotrophic factor/TrkB axis was activated in BDNF‐treated rat hearts.

### Effects of BDNF on morphological changes of ventricular myocytes in Dox‐treated rats

**Figure 2:**
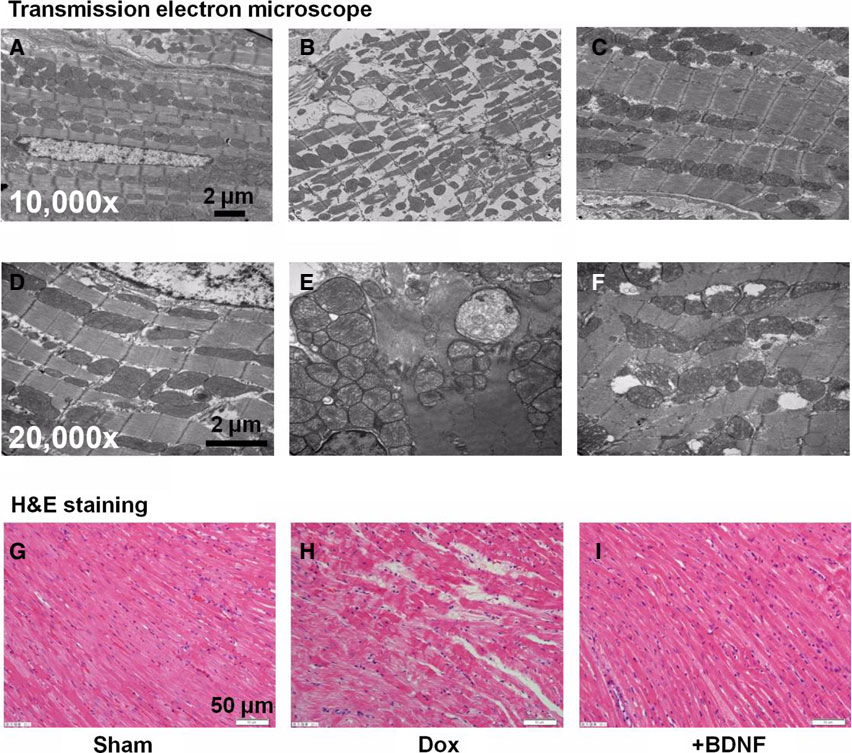
Morphological alterations of LV myocytes in Dox and BDNF‐treated rats. (**A**–**F**) Transmission electron microscope images showed the ultrastructure changes of LV myocytes of sham, Dox and BDNF‐treated rats, at magnification 10,000× and 20,000×, respectively, scale bar: 2 μm. (**G**–**I**) Representative images of haematoxylin and eosin staining from left ventricles of sham, Dox and BDNF‐treated rats, at magnification 400×, scale bar: 50 μm.

Transmission electron microscopy and haematoxylin and eosin staining were employed to examine the ultrastructural and histological changes of LV myocytes in Dox and BDNF‐treated rats. As shown in Figure 2A and D, a clear regular structure was observed in the cardiac muscle fibres of sham rats at different magnifications. However, disorganized myofibres and swollen mitochondria were shown in Dox‐treated cardiomyocytes (Fig. 2B and E), which was markedly improved by BDNF (Fig. 2C and F). Similarly, haematoxylin and eosin staining results displayed compactly arranged fibres without inter‐cellular space in cardiomyocytes of sham rats (Fig. 2G). In contrast, disordered myocardial fibres as well as hypertrophic and oedematous cardiomyocytes were observed in the Dox‐treated rats (Fig. 2H), which was significantly reversed by BDNF (Fig. 2I). Thus, these findings together displayed that BDNF mitigated Dox‐induced cardiac injury in rat ventricular myocytes.

### Effects of BDNF on cardiac apoptosis and fibrosis in Dox‐treated rats

**Figure 3:**
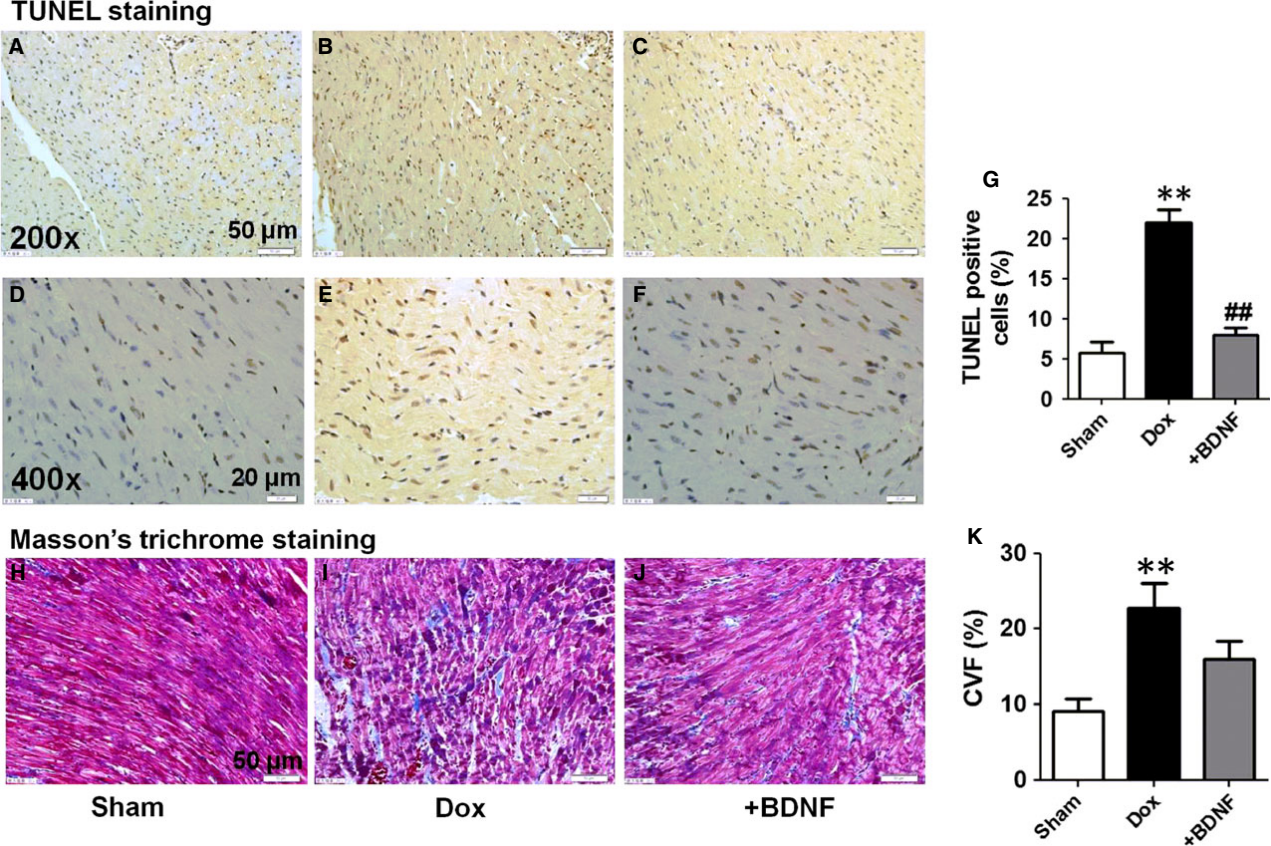
Cardiac apoptosis and interstitial fibrosis of left ventricles in Dox and BDNF‐treated rats. (**A**–**F**) TUNEL staining, the images at magnification 200× (scale bar: 50 μm) and 400× (scale bar: 20 μm). (**G**) Ratio of TUNEL‐positive cells (stained in brown) per field. (**H**–**J**) Representative Masson trichrome staining images from left ventricles of sham, Dox and BDNF‐treated rats. The images were magnified 400×, scale bar: 50 μm. (**K**) Collagen volume fraction was calculated as the ratio of collagen area to total area. **P < 0.01 *versus* sham; ##*P* < 0.01 *versus D*ox, *n* = 5 each group.

We further detected cardiomyocyte apoptosis and interstitial fibrosis in Dox and BDNF‐treated rats. As shown in Figure 3A–F, TUNEL assay was used to detect the effect of BDNF on cardiac apoptosis in Dox‐treated rat left ventricles at different magnifications. TUNEL‐positive cells in left ventricles were significantly higher in the Dox‐treated rats than sham rats, which was markedly reduced by BDNF (Fig. 3G). In addition, the cardimyocytes of sham rats appeared grossly normal under light microscopy. However, obvious interstitial fibrosis was examined in Dox‐treated rats. Bundles of myofibres were packed less tightly and were separated by thick layers of fibrous tissue in Dox‐treated rats, which was slightly reversed by BDNF (Fig. 3H–J). Statistically, CVF was significantly elevated in Dox‐treated rats compared to sham rats but did not significantly changed by BDNF (Fig. 3K). Consistently, the effects of Dox and BDNF on cardiac apoptosis and fibrosis in right ventricles were similar to left ventricles (Fig. S1).

### Effects of BDNF on oxidative stress and cardiac function in Dox‐treated rats

**Figure 4:**
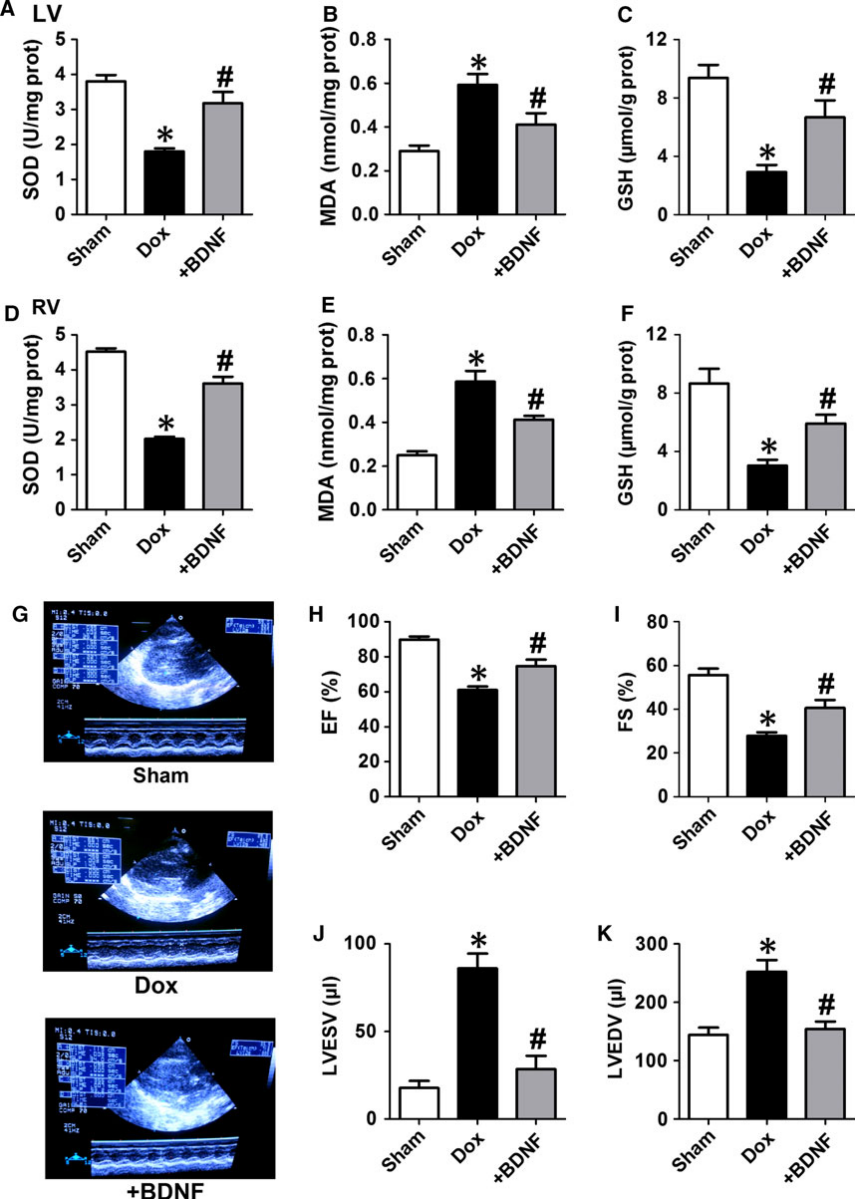
Effect of BDNF on oxidative stress and cardiac function of Dox‐treated rats by echocardiography. (**A** and **D**) Superoxide dismutase (SOD) activity, (**B** and **E**) malondialdehyde (MDA) level and (**C** and **F**) reduced glutathione (GSH) content in left and right rat ventricles. (**G**) Representative images of cardiac function. (**H**) Ejection fraction (EF). (**I**) Fractional shortening (FS). (**J**) LV end systolic volume (LVESV). (**K**) LV end diastolic volume (LVEDV). *P < 0.05 *versus* sham, #*P* < 0.05 *versus* Dox, *n* = 5 each group.

It is well known that SOD, MDA and reduced GSH are good indicators of oxidative stress. Therefore, these markers are examined to assess the effects of BDNF on oxidative stress in our study. It was found that BDNF significantly restored antioxidant markers (SOD activity and GSH content) and decreased MDA level in Dox‐treated left and right ventricles (Fig. 4A–F). We further validated the salutary role of BDNF in protection of Dox‐induced cardiac dysfunction. Echocardiographic data indicated that BDNF significantly ameliorated cardiac function in Dox‐treated rats (Fig. 4G). In detail, BDNF markedly increased LVEF and FS (Fig. 4H and I), as well as decreased LVESV and LVEDV (Fig. 4J and K). Therefore, the above findings strongly supported that BDNF protected against Dox‐induced oxidative stress and cardiac dysfunction.

### Effects of BDNF on protein expression of Akt signals in Dox‐treated rat ventricles

**Figure 5:**
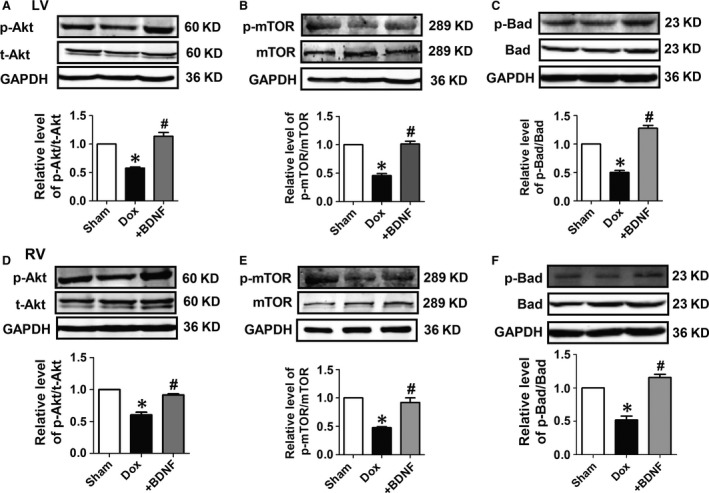
Protein expression of Akt signalling in Dox and BDNF‐treated rats. (**A** and **D**) Representative western blot bands and statistical results of phosphorylated Akt (p‐Akt) and total Akt (t‐Akt) of left and right ventricles with GAPDH as internal control. (**B** and **E**) Representative western blot bands and statistical results of phosphorylated mammalian target of rapamycin (p‐mTOR) and mTOR of left and right ventricles. (**C** and **F**) Representative western blot bands and statistical results of phosphorylated Bad (p‐Bad) and Bad of left and right ventricles. *P < 0.05 *versus* sham, #*P* < 0.05 *versus* Dox, *n* = 5 each group.

We then examined the role of Akt signals in the anti‐apoptotic effect of BDNF against Dox‐induced cardiac injury in both left and right ventricles. It was found that protein level of p‐Akt was significantly repressed by Dox, which was recovered by BDNF (Fig. 5A) in left rat ventricles. Meanwhile, no significant change in t‐Akt was observed among these groups (Fig. 5A). We further detected protein expression of mTOR and Bad, the downstream signal of PI3K/Akt pathway, and found that p‐mTOR and p‐Bad were inhibited by Dox and was restored by BDNF. In addition, mTOR and Bad protein levels did not change in those three groups (Fig. 5B and C). Consistently, similar results were found in right ventricles (Fig. 5D–F). So, these data supported that Akt regulated mTOR and Bad signals were involved in the protective effect of BDNF against Dox‐induced cardiotoxicity.

### Effects of BDNF on protein expression of p38 MAPK and ERK in Dox‐treated rat ventricles

**Figure 6:**
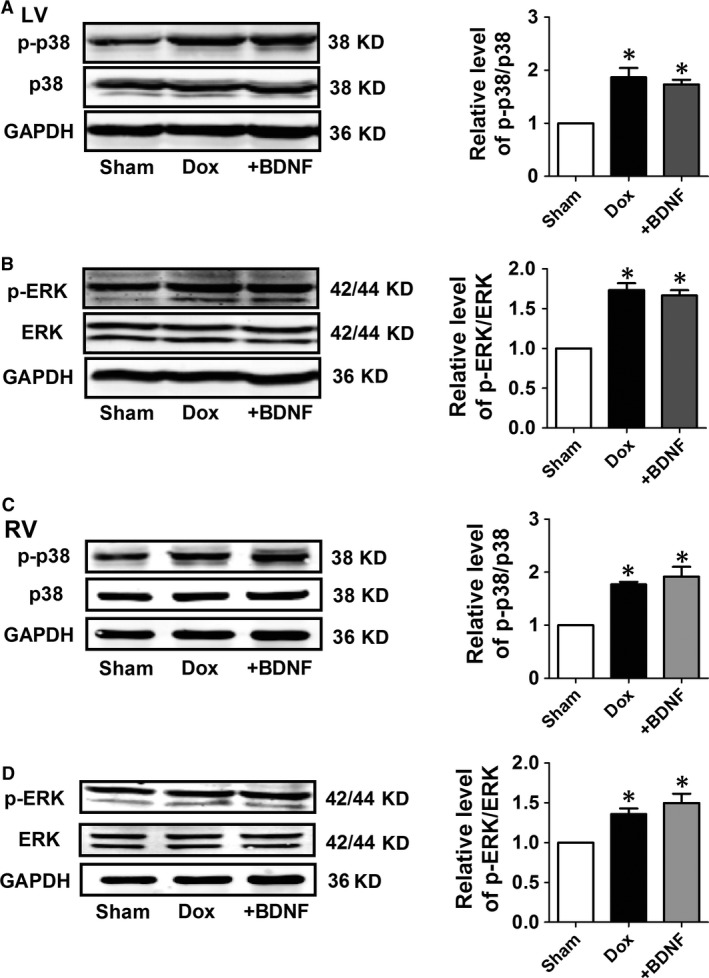
Protein expression of p38 and ERK in Dox and BDNF‐treated left and right rat ventricles. (**A** and **C**) Representative western blot bands and statistical results of p‐p38 and p38 of left and right ventricles. (**B** and **D**) Representative western blot bands and statistical results of p‐ERK and ERK of left and right ventricles. *P < 0.05 *versus* sham, *n* = 5 each group.

Mitogen‐activated protein kinase family members including p38 MAPK and ERK were also examined. We found that protein levels of p‐p38 and p‐ERK as well as ratios of p‐p38/p38 and p‐ERK/ERK were increased in Dox rats, however, did not significantly change after BDNF treatment (Fig. 6A and B). Similarly, expression of p38 MAPK and ERK in right ventricles are consistent with findings in left ventricles (Fig. 6C and D).

### Effects of BDNF on cell viability of Dox‐treated H9c2 cells

**Figure 7:**
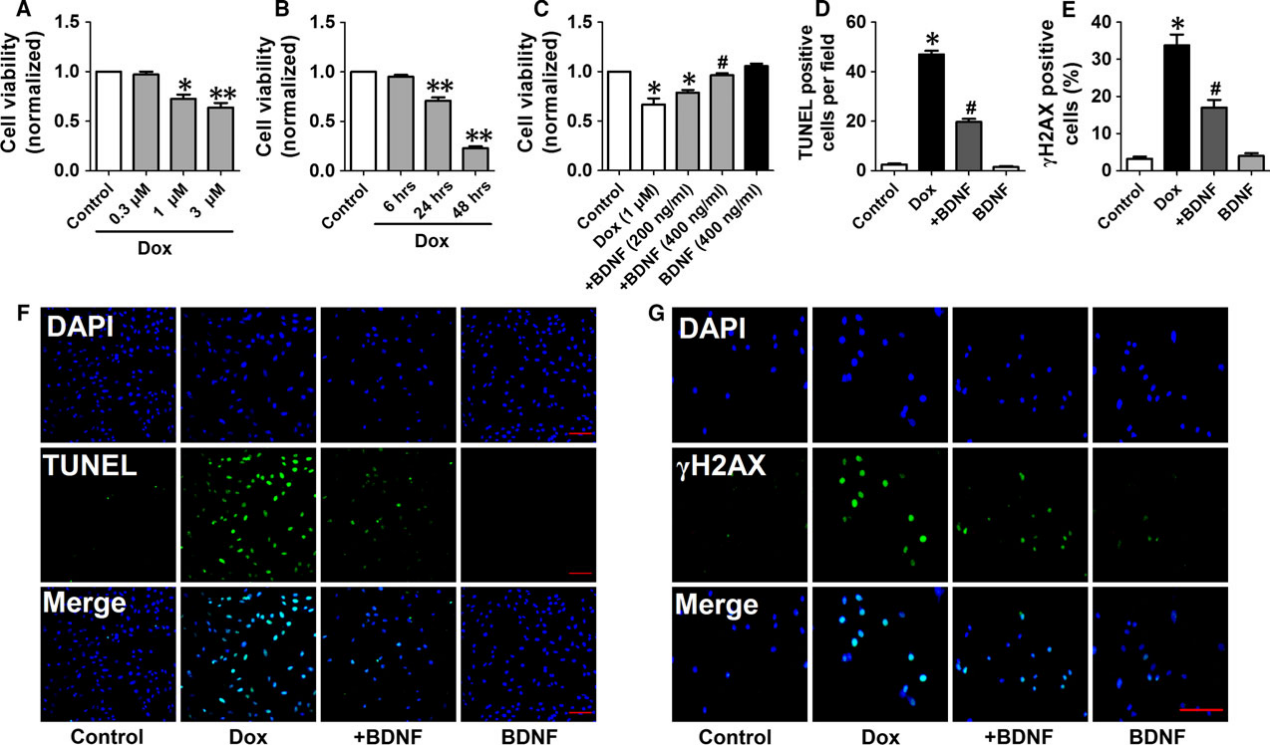
BDNF antagonized Dox‐induced toxicity of H9c2 cells. (**A**) Effect of different concentrations of BDNF including 0.3, 1, 3 μM for 24 hrs on cell viability of H9c2 cells. (**B**) Effect of different durations of BDNF including 6, 24 and 48 hrs at 1 μM on cell viability of H9c2 cells. (**C**) Effect of BDNF (200, 400 ng/ml) on Dox (1 μM)‐treated cell toxicity of H9c2 cells. (**D**) BDNF reduced apoptotic rate in Dox‐induced H9c2 cells. (**E**) BDNF decreased γH2AX positive cells in Dox‐induced H9c2 cells. (**F**) Representive images of TUNEL staining showing the apoptotic cells (stained in green) and nucleus (stained in blue with DAPI), scale bar: 100 μM. (**G**) Representive images of γH2AX staining showing the DNA damage (stained in green) and nucleus (stained in blue with DAPI), scale bar: 100 μM. *P < 0.05, **P < 0.01, *versus* control, #*P* < 0.05 *versus* Dox, *n* = 5 each group.

Then, we aimed to validate the effect and mechanisms of BDNF on the cardiotoxicity of Dox *in vitro*. First, we examined the dose–response of Dox in H9c2 cells. As shown in Figure 7A, the cell viability was markedly reduced by Dox at 1 and 3 μM for 24 hrs, whereas not significantly changed by 0.3 μM Dox. Then, we tested the various time‐points and found that Dox (1 μM) treatment for 24 and 48 hrs markedly reduced the cell viability, however, 6 hrs treatment did not significantly reduced the cell viability (Fig. 7B). Accordingly, 1 μM Dox treatment for 24 hrs were selected for subsequent experiments. Furthermore, we detected the effect of BDNF on cell viability treated with Dox. We found that BDNF (400 ng/ml) significantly reversed cell viability induced by Dox (Fig. 7C). Therefore, BDNF (400 ng/ml) was determined for the following studies.

### Effects of BDNF on apoptosis and DNA damage in Dox‐treated H9c2 cells

**Figure 8:**
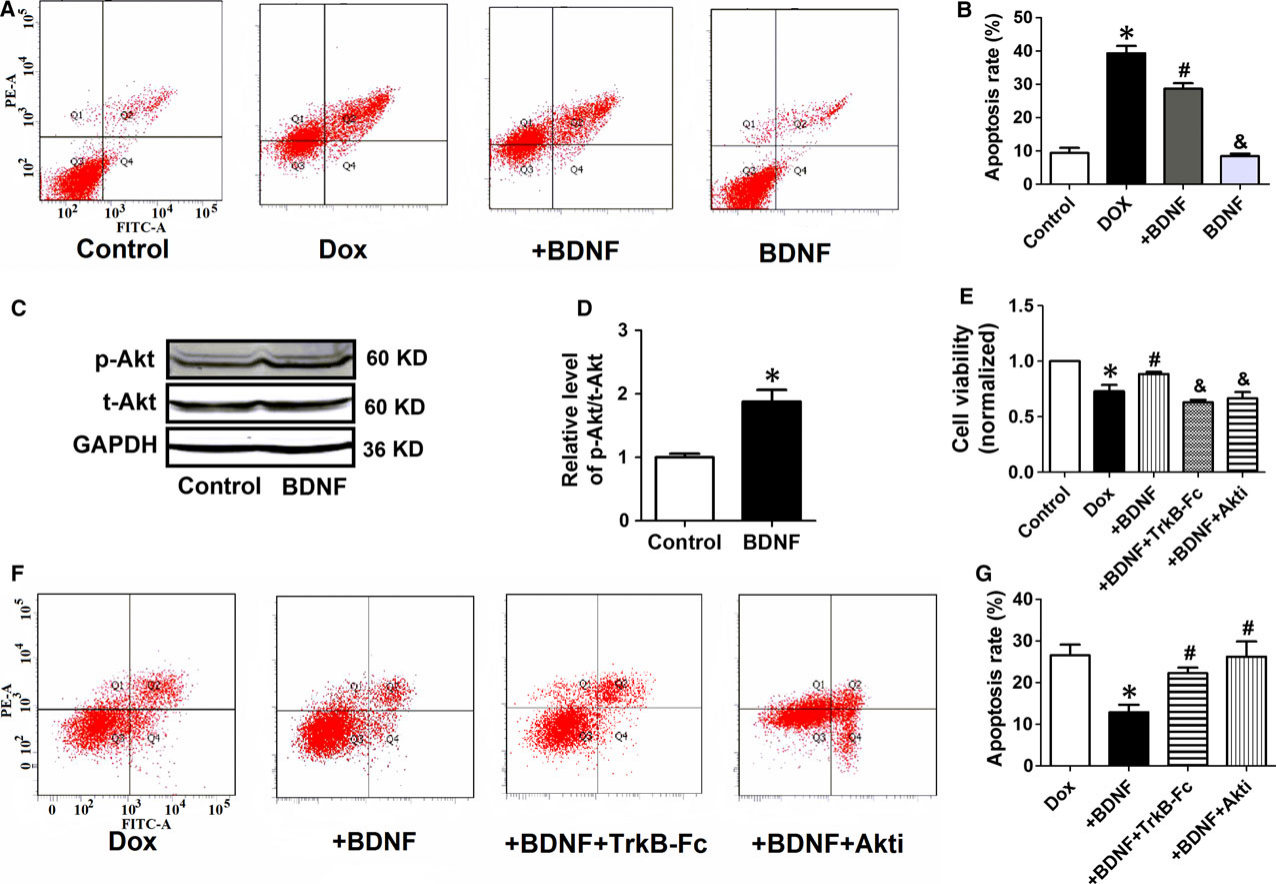
BDNF inhibited Dox‐induced apoptosis of H9c2 cells by activating Akt. (**A**) Annexin V‐FITC/propidium iodide (PI) staining by flow cytometry was performed to detect the apoptosis of H9c2 cells. Early apoptosis (Annexin V‐FITC ^+^/PI ^−^, Q4), late apoptosis (Annexin V‐FITC ^+^/PI ^+^, Q2), and necrosis (Annexin V‐FITC ^−^/PI ^+^, Q1). (**B**) The quantitative presentation of apoptotic cell population by Annexin V‐FITC/PI staining. *P < 0.05 *versus* control, #*P* < 0.05 *versus* Dox, &*P* < 0.05 *versus* +BDNF,* n* = 3 each group. (**C**) Western blot analysis of the p‐Akt and t‐Akt. GAPDH served as the internal control. (**D**) Statistical results of p‐Akt/t‐Akt in H9c2 cells. *P < 0.05 *versus* control, *n* = 5 each group. (**E**) Effect of TrkB‐Fc and Akti on cell viability of BDNF‐treated H9c2 cells. *P < 0.05 *versus* control, #*P* < 0.05 *versus* Dox, and *P* < 0.05 *versus* +BDNF,* n* = 5 each group. (**F**) The quantitative presentation of apoptotic cell population by Annexin V‐FITC/PI staining. (**G**) Annexin V‐FITC/PI staining by flow cytometry was performed to detect the apoptosis. *P < 0.05 *versus* Dox, #*P* < 0.05 *versus* +BDNF,* n* = 3 each group.

TUNEL assay and flowcytometry were employed to confirm the anti‐apoptotic effect of BDNF in Dox‐treated H9c2 cells. It was suggested that TUNEL‐positive cells dramatically increased in Dox group, which was reversed by BDNF (Fig. 7D and E). Phosphorylated γH2AX, a sensitive marker of double‐stranded DNA break, was employed to assess whether BDNF reduced DNA damage in Dox‐treated H9c2 cells (Fig. 7F). Fluorescence intensity of γH2AX was higher in Dox group than untreated cells, which was limited by BDNF (Fig. 7G). Furthermore, as shown in Figure 8A, the apoptotic rate was increased by Dox, which was attenuated by BDNF (Fig. 8B). Taken together, these findings supported that BDNF protected against Dox‐induced apoptosis in H9c2 cells.

### TrkB‐Fc and Akti abolished the protective role of BDNF in Dox‐treated H9c2 cells

We further confirmed the impact of BDNF on protein expression of Akt in H9c2, and found that BDNF treatment significantly increased p‐Akt level without affecting t‐Akt level (Fig. 8C and D), which is agreement with previous *in vivo* results. Then, BDNF blocker TrkB‐Fc and Akti were employed to validate the pivotal role of BDNF/TrkB/Akt cascade in beneficial effect of BDNF. The cell viability in BDNF‐treated cells was reversed by BDNF scavenger TrkB‐Fc or Akti (Fig. 8E). It was also found that the anti‐apoptotic effect of BDNF against Dox was significantly restored by TrkB‐Fc or Akti (Fig. 8F and G). Collectively, our findings suggest that Akt signal is indispensible for the cardioprotective effect of BDNF against Dox‐induced cardiotoxicity.

## Discussion

This study mainly found that (*i*) cardiac BDNF expression was downregulated in Dox‐treated rat; (*ii*) exogenous BDNF inhibited Dox‐induced cardiac dysfunction including oxidative stress, DNA damage and cardiomyocyte apoptosis by activating Akt signalling and preserving phosphorylation of mTOR and Bad; (*iii*) BDNF did not affect p38 MAPK and ERK in Dox‐treated rat heart.

In our previous study, it was found that protein expression of BDNF was increased in response to acute ischaemia for 1 or 6 hrs 19. On the contrary, other study reported that chronic myocardial ischaemia (range from 1 to 14 days) significantly decreased protein level of BDNF with time‐dependent manner 15. Besides, plasma BDNF level was elevated in acute myocardial infarction patients 19 while reduced in heart failure patients 31, 32. On the basis of these findings, we speculated that increment of BDNF expression in acute injury was a compensatory mechanism while reduction expression after sustained injury was the outcome of decompensation. In agreement with the above findings, we found that protein level of BDNF in the myocardium of Dox‐treated rats was markedly decreased. Exogenous BDNF not only reversed cardiac BDNF expression but also improved Dox‐induced cardiac dysfunction. Accordingly, BDNF deficiency is probably the consequence of Dox‐induced cardiotoxicity, which was restored after BDNF treatment.

It is well known that TrkB is the high‐affinity receptor of BDNF, which is indispensable for functional role of BDNF in both neurons 33 and heart 16, 17. Our previous study also confirmed the crucial role of BDNF/TrkB pathway in the heart 19. In this study, we observed the protective effect of BDNF against Dox‐induced cardiomyocyte apoptosis was recovered by TrkB‐Fc. In addition, TrkB could induce PI3K activity and leads to Akt activation by phosphorylating Akt at ser473 34. We demonstrated the BDNF/TrkB/Akt axis in the protection of Dox‐induced cardiac injury. Once Akt is activated, the phosphorylation of downstream key molecules mTOR and Bad are up‐regulated and regulates many important cellular activities including proliferation, growth, survival and angiogenesis. PI3K/Akt pathway was well recognized to promote survival by downregulating pro‐apoptotic factors and up‐regulating anti‐apoptotic factors. Through phosphorylation, PI3K/Akt inhibits the activity of proapoptotic members while activating anti‐apoptotic members 35. On the other hand, MAPK family members p38 MAPK and ERK have also been demonstrated participated in Dox‐induced cardiac dysfunction 26. In our present study, both p‐p38 MAPK and p‐ERK were activated by Dox, however, neither of them was affected by BDNF. So, p38 MAPK and ERK were not involved in the protective role of BDNF against Dox‐induced injury.

Consistently, it has been previously described with effects of BDNF against Dox‐induced apoptosis in cancer cells 36. Middlemas *et al*. reported the beneficial role of BDNF against cytotoxic agents including cisplatin, Dox and topotecan 20. They found that BDNF protected cell against DNA damaging caused by these agents. Moreover, they also speculated that BDNF regulate a common signalling pathway which is required for cell death 20. Similarly, Ho *et al*. revealed that BDNF/TrkB signalling pathway has a beneficial impact when exposed to DNA‐damaging reagents in neuroblastomas. Moreover, they also found that activation of PI3K/Akt survival pathway may contribute to the increased Dox resistance in TrkB‐expressing neuroblastomas 21. Lately, another study reported that BDNF has potent cytoprotective effects against Dox‐induced injury in pancreatic β cells 37. These findings suggest that the protective effect of BDNF may exist in different types of cells.

PI3K/Akt pathway phosphorylates many intracellular substrates 38. It has been revealed that BDNF exerted its pro‐survival effects by binding TrkB and activating downstream signalling pathways. In particular, TrkB‐induced PI3K activity lead to Akt activation and thereby phosphorylated downstream pro‐apoptotic targets, including Forkhead and Bad2 39, 40. Moreover, up‐regulation of BDNF activated Akt and promotes hippocampal neuronal proliferation in mice by regulating Bcl‐2 and caspase‐3 41. Combined ampakine and local delivery of BDNF improved post‐stroke functional recovery in aged mice by activating Akt/CREB signalling 42. Here, we validate that phosphorylation of mTOR and Bad are preserved by BDNF in Dox‐induced cardiotoxicity. Of course, other signals modulated by BDNF against Dox‐induced cardiac injury could not be excluded, although p38 and ERK were determined. But the present findings strongly suggest that Akt signals are central in the protection of BDNF against Dox‐induced cardiac injury. On the other hand, although BDNF has promising effect on cardiomyocyte injury, it should be noted that Dox‐induced interstitial fibrosis was not significantly ameliorated by BDNF. Thus, whether BDNF reverses cardiac remodeling in several periods of Dox treatment is interesting to study in the following work.

In summary, the findings of this study highlight potential implications of BDNF against Dox‐induced cardiac dysfunction, which may enhance our understanding of therapeutic role of neurotrophic factors against chemotherapeutic agents induced cardiotoxicity to facilitate their safe use in cancer treatment.

## Conflict of interest

The authors declare that they have no conflicts of interest.

## Supporting information


**Figure S1** Cardiac apoptosis and interstitial fibrosis of right ventricles in Dox and BDNF‐treated rats.
