# Supplementary material for: Brain‐derived neurotrophic factor attenuates doxorubicin‐induced cardiac dysfunction through activating Akt signalling in rats
Source: J Cell Mol Med. 2016 Nov 7;21(4):685–96. doi: 10.1111/jcmm.13012 (PMC5345637; doi:10.1111/jcmm.13012)
Supplement: Supplementary file 1 — Figure S1 Cardiac apoptosis and interstitial fibrosis of right ventricles in Dox and BDNF‐treated rats. [file JCMM-21-685-s001.doc]

**Brain-derived neurotrophic factor attenuates doxorubicin-induced cardiac dysfunction through activating Akt signaling in rats**

**Supplemental Figure and Figure Legend**


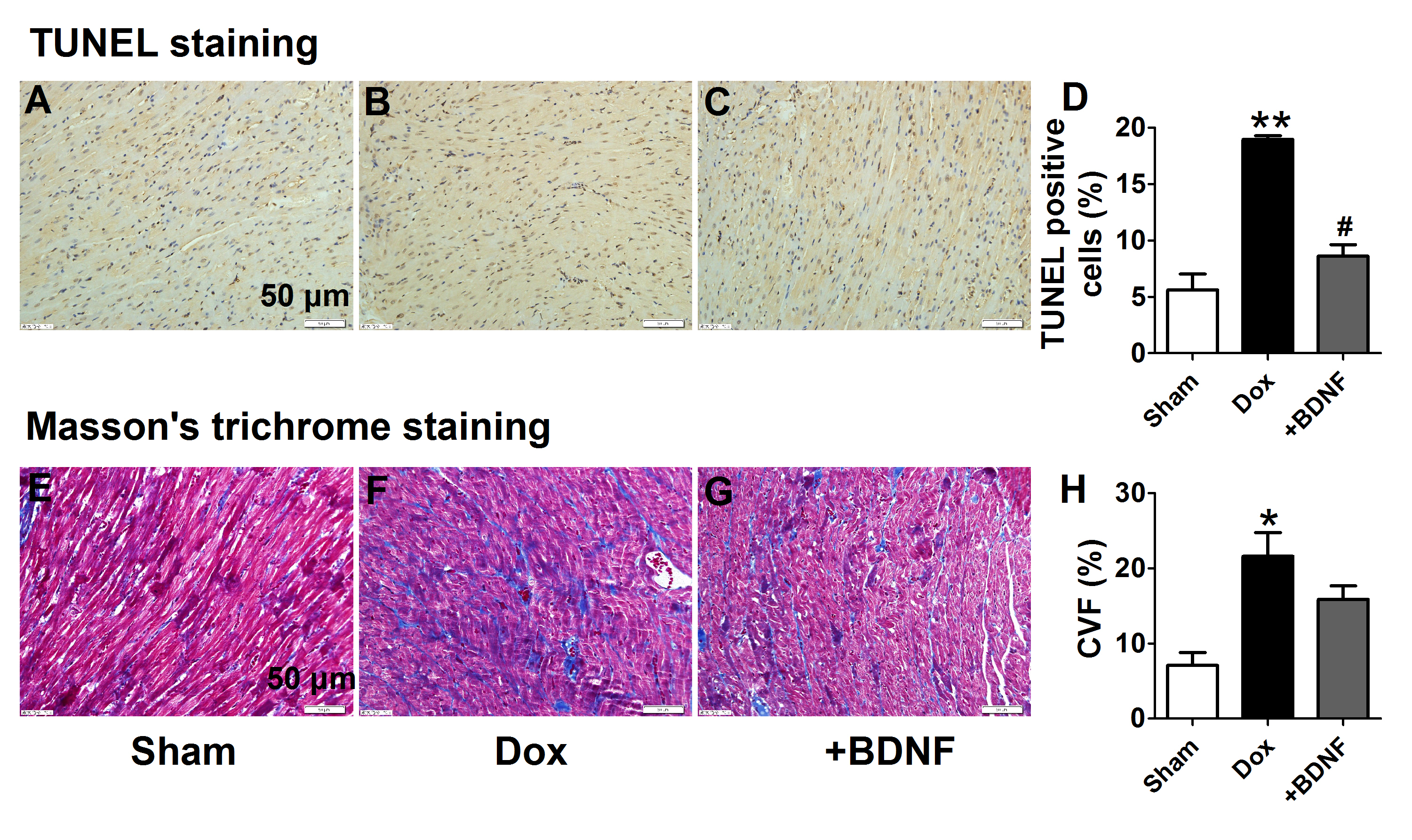


Supplemental Figure 1 Cardiac apoptosis and interstitial fibrosis of right ventricles in Dox and BDNF-treated rats. **P* < 0.05, ***P* < 0.01 vs. sham, #*P* < 0.05 vs. Dox, n = 5 each group, scale bar: 50 μm.
